# Highlighting mass spectrometric fragmentation differences and similarities between hydroxycinnamoyl-quinic acids and hydroxycinnamoyl-isocitric acids

**DOI:** 10.1186/s13065-017-0262-8

**Published:** 2017-04-04

**Authors:** Keabetswe Masike, Msizi I. Mhlongo, Shonisani P. Mudau, Ofentse Nobela, Efficient N. Ncube, Fidele Tugizimana, Mosotho J. George, Ntakadzeni E. Madala

**Affiliations:** 1 Department of Biochemistry, University of Johannesburg, Auckland Park, P.O. Box 524, Johannesburg, 2006 South Africa; 2 Department of Chemistry and Chemical Technology, National University of Lesotho, P.O. 180, Roma, Lesotho

**Keywords:** *Amaranthus viridis*, Hydroxyl-cinnamic acid, Hydroxycinnamoyl-isocitric acid, Hydroxycinnamoyl-quinic acid, Mass spectrometry, *Moringa oleifera*

## Abstract

**Background:**

Plants contain a myriad of metabolites which exhibit diverse biological activities. However, in-depth analyses of these natural products with current analytical platforms remains an undisputed challenge due to the multidimensional chemo-diversity of these molecules, amplified by both isomerization and conjugation. In this study, we looked at molecules such as hydroxyl-cinnamic acids (HCAs), which are known to exist as positional and geometrical isomers conjugated to different organic acids namely quinic- and isocitric acid.

**Objective:**

The study aimed at providing a more defined distinction between HCA conjugates from *Amaranthus viridis* and *Moringa oleifera*, using mass spectrometry (MS) approaches.

**Methods:**

Here, we used a UHPLC–MS/MS targeted approach to analyze isobaric HCA conjugates extracted from the aforementioned plants.

**Results:**

Mass spectrometry results showed similar precursor ions and fragmentation pattern; however, distinct differences were seen with ions at *m/z* 155 and *m/z* 111 which are associated with isocitric acid conjugates.

**Conclusion:**

Our results highlight subtle differences between these two classes of compounds based on the MS fingerprints, enabling confidence differentiation of the compounds. Thus, these findings provide a template reference for accurate and confident annotation of such compounds in other plants.

**Electronic supplementary material:**

The online version of this article (doi:10.1186/s13065-017-0262-8) contains supplementary material, which is available to authorized users.

## Background

Plants are a source of various natural compounds with a wide spectrum of bioactivities. These compounds are categorized into primary and secondary metabolites, where the former are involved in housekeeping functions and the latter are used by plants in interactions with their environment [1]. The most dominant of the secondary metabolites are phenylpropanoids, a class of compounds that bear a 3-carbon (C-3) chain linked to 6-carbon (C-6) aromatic ring [2–5]. The diversification of phenylpropanoids in different plant species has previously been attributed to the presence or absence of active enzymes involved in their biosynthetic pathway [2, 6]. Some of the known phenylpropanoids include flavonoids, isoflavonoids, coumarins, anthocyanins, stilbenes, benzoic acids, benzaldehyde derivatives, phenylpropenes and hydroxyl-cinnamic acid (HCA) derivatives, among others [2, 7, 8]. HCA derivatives form one of the largest classes of phenylpropanoid-derived plant compounds [9, 10], and include caffeic-, ferulic- and *p*-coumaric acids. These metabolites contribute to the abundance of plant natural products as they form conjugates with different molecules such as sugars, polyamines and organic acids [9, 11–15]. The most common example of HCAs conjugated to organic acids are chlorogenic acids (CGAs), which are formed from an esterification reaction between the organic acid, quinic acid (QA) and one to four (identical or different) residues of HCA derivatives [12].

**Scheme 1:**
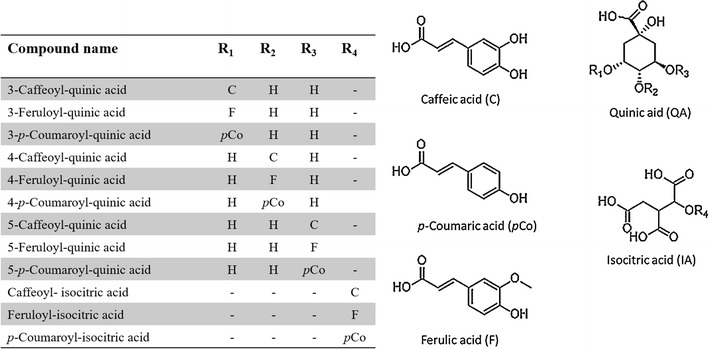
Structures of mono-acylated HCA conjugates of quinic and isocitric acid

In nature, *mono*-acyl CGAs commonly occur as three *regio*-isomers where C3, C4 and C5 hydroxides on the QA are esterified giving rise to three positional isomers [16–18]. However, 1-acyl CGA has occasionally been noted in some plant species [19, 20]. Lastly, geometrical isomerization (*trans* and *cis*) of the different HCA derivatives seals the final diversification of these molecules [14–17, 21–24]. Another example of HCA derivatives forming conjugates with organic acids includes the esterification between isocitric acid (IA) and one of the HCA derivatives to form hydroxycinnamoyl-isocitric acid [25] as shown in Scheme 1. Unlike QA with four possible esterification positions, this esterification of IA moiety can occur at position 2 (C2). In addition, the diversification of hydroxycinnamoyl-isocitric acid only includes the conjugation of different HCA derivatives to the organic acid and the geometrical isomerization thereof. The botanical distribution of hydroxycinnamoyl-isocitric acid derivatives is not well documented. This is possibly due to the misidentification as *mono*-acyl CGAs since both respective group of compounds have a molecular mass of 354 Da for caffeoyl-, 338 Da for *p*-coumaroyl- and 368 Da for feruloyl conjugates [16, 25].

In recent years, liquid chromatography (LC)–MS has become one of the most common techniques for annotation of plant metabolites as well as discerning between different positional isomers of *mono*-, *di*- and *tri*-acyl CGAs [14–16, 22, 23, 26, 27]. However, very little has been done for geometrical isomers of CGAs [28, 29]. Despite the remarkable analytical developments and methodologies, there are still some common misrepresentation in annotation of these two classes of compounds. This could be due to their similar MS fragmentation patterns leading to poor resolution and un-differentiation of these molecules thereafter. Herein we, demonstrate the unique and similar chromatographic and mass spectrometric characteristics of hydroxycinnamoyl-quinic- and hydroxycinnamoyl-isocitric acids using LC–MS experiments. Authentic standards and plant extracts of *Moringa oleifera* and *Amaranthus viridis*, were employed to demonstrate the common elements that bring confusion. These two plant species are reported to respectively accumulate/produce these compounds in abundance [24, 30].

## Methods

### Chemical and reagents

Authentic standards of caffeic acid-derived chlorogenic acids (3-, 4- and 5-caffeoylquinic acid) were purchased from Phytolab (Vestenbergsgreuth, Germany). Analytical-grade methanol and acetonitrile were purchased from Romil Pure Chemistry (Cambridge, UK). Formic acid was obtained from Sigma-Aldrich (St. Louis, MO, USA).

### Metabolite extraction

The dried leaves of *M. oleifera* and *A. viridis* were pulverized using a clean and dry quartz mortar and pestle. For extraction, the respective amounts of powdered leaf material (0.2 g) were mixed with 2 mL of 50% aqueous methanol and these extracts were placed (with the lids of the tubes closed to avoid evaporation) in a heating block at 60 °C for 2 h. The samples were sonicated for 30 min using an ultrasonic bath and then centrifuged at 9740×*g* for 10 min at 4 °C. The resulting supernatants for both plant samples were then subjected to UV-irradiation for induction of geometrical isomerization [21]. Coffee bean- and pineapple extracts to be used as surrogate standards were prepared by extracting 0.2 g of these materials in 1 mL of 50% methanol.

### Ultra-high performance liquid chromatography mass spectrometry (UHPLC–MS/MS) analysis

A Shimadzu Nexera 30 UHPLC (Kyoto Japan) fitted with a Viva C_18_ analytical column (3.0 µm, 2.1 × 100 mm; Restek, USA) was used with the following settings: an injection volume of 2 µL, column oven temperature of 40 °C, a binary solvent mixture consisting of MilliQ water containing 0.1% formic acid (eluent A) and methanol containing 0.1% formic acid (eluent B) with a constant flow rate of 0.4 mL/min. The gradient elution was used with the following conditions: 5% eluent B maintained for 3 min, followed by a linear increase to 45% of eluent B at 25 min, then a further increase to 90% at 30 min, conditions were held constant for 2 min before being decreased to the initial conditions at 34 min followed by a 6 min isocratic wash at 5% to re-equilibrate the column. The total chromatographic run time was 40 min. The data were acquired using a UV detector set at 325 nm.

The chromatographic effluent was further introduced to an MS detector and ionized by electrospray (ESI). The ionized ions were further analyzed by a triple quadrupole (QqQ) mass spectrometer operating under the following settings: the interface voltage was set at 3.5 kV (in negative ESI mode), the source temperature was 300 °C, nitrogen was used as the drying gas at the flow rate of 15.00 L/min and argon used as a nebulizing gas at a flow rate of 3.00 L/min, argon was also used as a collision gas with a pressure of approximately 230 kPa in the collision cell. For each run, the MS spectra at the mass range 100–1000 Da was collected continuously with a scan time of 1 s. For targeted analyses, the product scan MS mode was used to monitor the fragmentation patterns of the following ions: *m/z* 353 for caffeoyl-quinic acid and caffeoyl-isocitric acid, *m/z* 337 for coumaroyl-quinic acid and coumaroyl-isocitric acid and finally *m/z* 367 for feruloyl-quinic acid and feruloyl-isocitric acid. Exhaustive MS fragmentation was achieved by collecting data at various collision energies (5–35 eV) to mimic MS^E^ experiments.

## Results and discussion

### Compound annotation

**Fig. 1:**
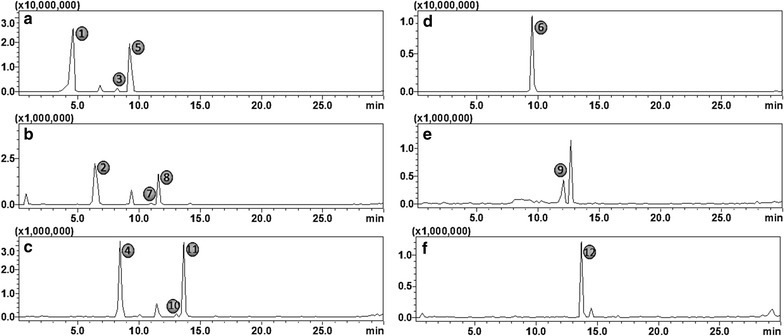
UHPLC–SIM-MS chromatograms of selected HCA conjugates from *M. oleifera* (**a**–**c**) and *A. viridis* extracts (**d**–**f**). HCAs conjugated to quinic acid: **a** caffeoyl-quinic acids, **b** *p*-coumaroyl-quinic acid and **c** feruloyl-quinic acid. HCAs conjugated to isocitric acid: **d** caffeoyl-isocitric acid, **e** *p*-coumaroyl-isocitric acid and **f** feruloyl-isocitric acid

As one of the main aspects of the present study, we compare hydroxycinnamoyl-quinic- and hydroxycinnamoyl-isocitric acid derivatives and show how both chromatography and mass spectrometry can be used to distinguish these isobaric compounds. Single ion monitoring (SIM) chromatograms of hydroxycinnamoyl-quinic- and hydroxycinnamoyl-isocitric acid from *M. oleifera* and *A. viridis* leaf extracts are shown respectively in Fig. 1. The mass spectra and retention times of the compounds under study were compared with those of available standards (i.e. 3-CQA, 4-CQA and 5-CQA). Coffee bean extracts have been previously reported to be remarkably rich in a variety of CGAs, including feruloyl and *ρ*-coumaroyl derivatives [9, 13, 27]. Furthermore, a study by Steingass et al. [31] revealed the presence of hydroxycinnamoyl isocitric acids in pineapple extracts. Hence in this study, coffee bean- and pineapple extracts were analyzed using the same optimized method and the results obtained therefore served as surrogate standards for feruloyl and *ρ*-coumaroyl- and IA derivatives, respectively (Additional file 1: Figure S1).

**Fig. 2:**
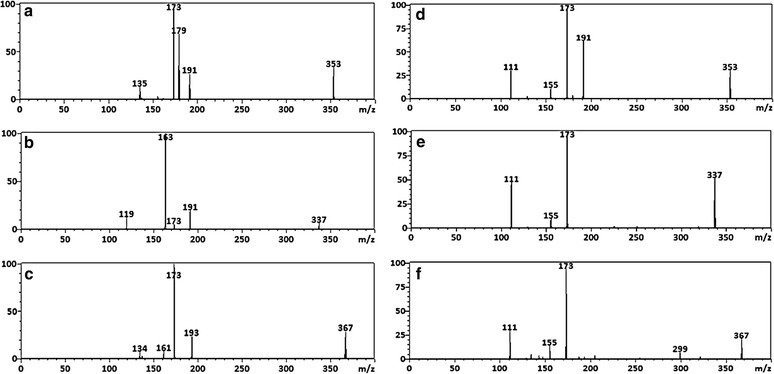
Typical MS fragmentation patterns of HCAs conjugated to quinic acid (**a**–**c**) extacted from *M. oleifera* or isocitric acid (**d**–**f**) extracted from *M. viridis*: **a** 4-caffeoyl-quinic acid, **b** 4-*p*-coumaroyl-quinic acid, **c** 4-feruloyl-quinic acid, **d** 2-caffeoyl-isocitric acid, **e** 2-*p*-coumaroyl-isocitric acid and **f** 2-feruloyl-isocitric acid

In addition, the annotation of hydroxycinnamoyl-quinic- and hydroxycinnamoyl-isocitric- acids was also achieved by comparing MS fragmentation patterns with those of commercially available standards (Fig. 2). HCA conjugates of both QA **(**Fig. 2a–c) and IA (Fig. 2d–f) are isobaric and produce precursor ion peaks at *m/z* 337, 353 and 367 for *p*-coumaroyl-, caffeoyl- and feruloyl conjugates, respectively in negative ionization. According to the hierarchical fragmentation scheme proposed by Clifford et al. [16] the annotation of 4-acyl CGA derivatives is indicated by the presence of an intense product ion peak at *m/z* 173 [16]. However, MS fragmentation patterns of all hydroxylcinnamoyl isocitric acids also produce a peak at *m/z* 173 (Fig. 2) and, as such, these compounds are often wrongly annotated.

**Scheme 2:**
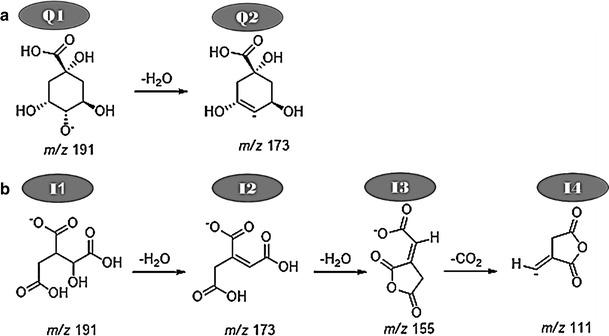
Main fragmentation mechanism and structural re-arrangement for the [M–H]^−^ ion of quinic acid (**a**) and isocitric acid (**b**) in negative ionization

Previous studies have pointed out several MS diagnostic peaks have been noted for HCA derivatives, where *p*-coumaric acid produces ions at *m/z* 163 [*p*-coumaric acid–H]^−^ and *m/*z 119 [*p*-coumaric acid–H–CO_2_]^−^ (observed also in our study in Fig. 2b), caffeic acid produces ions at *m/z* 179 [caffeic acid–H]^−^ and *m/*z 135 [caffeic acid–H–CO_2_]^−^ (observed also in our study in Fig. 2a) and ferulic acid produces ions at *m/z* 193 [ferulic acid–H]^−^ and *m/z* 134 [ferulic acid–H–CO_2_–CH_3_]^−^ (observed also in our study in Fig. 2c) [16, 23, 24]. However, one important observation/evidence emerging from this study is that these diagnostic patterns were only observed when HCA derivatives were attached to quinic acid (Fig. 2). This evidenced that the presence of HCA daughter peaks is a distinguishing character for quinic acid conjugates. Furthermore, in the current study, tandem MS (MS/MS) approach was used to distinguish between QA and IA derivatives. Given that both QA and IA have shown to produce similar MS spectra comprising of ions at *m/z* 191 and 173 in ESI negative mode (Scheme 2; Fig. 2a–f); this has subsequently led to the incorrect annotation of these molecules in some reported literature [28, 30]. Thus, to distinguish IA from the QA derivatives, the results obtained in this study revealed other diagnostic ion peaks at *m/z* 155 and 111 which were only observed for IA conjugates (Scheme 2; Fig. 2d–f), and these results are also consisted with published data shown elsewhere [25]. Hydroxycinnamoyl-quinic acid and hydroxycinnamoyl-isocitric acid structures are shown in Scheme 1 and the MS fragmentation patterns are summarized in Table 1.

**Table 1:** Characterization of hydroxyl-cinnamic acid conjugates from *M. oleifera* and *A. viridus*

No.	Rt (min)	Compound name	*M. oleifera*	*A. viridus*	[M–H]^−^ (*m*/*z*)	Fragmentations (*m*/*z*)
1	4.7	3-Caffeoyl-quinic acid	√		353	191, 179, 161, 135
2	6.3	3-*p*-Coumaroyl-quinic acid	√		337	191, 173, 163, 119
3	8.3	5-Caffeoyl-quinic acid	√		353	191, 135
4	8.5	3-Feruloyl-quinic acid	√		367	193, 191, 173, 149, 134
5	9.3	4-Caffeoyl-quinic acid	√		353	191, 179, 173, 135
6	9.6	Caffeoyl-isocitric acid		√	353	191, 173, 155, 111
7	11.1	5-*p*-Coumaroyl-quinic acid	√		337	191, 119
8	11.6	4-*p*-Coumaroyl-quinic acid	√		337	173, 163, 137, 119
9	12.2	*p*-Coumaroyl-isocitric acid		√	367	173, 155, 111
10	13.1	5-Feruloyl-quinic acid	√		337	191, 135
11	13.7	4-Feruloyl-quinic acid	√		367	193, 173, 134
12	13.9	*p*-Feruloyl-isocitric acid		√	367	173, 155, 111

Furthermore, in a chromatographic space, it was interestingly observed that IA derivatives elute later than the QA counterparts (Fig. 1 and Table 1). For example, all three CQA *regio*-isomers eluted at retention times (Rt) 4.7 min for 3-CQA, 8.3 min for 5-CQA and 9.3 min for 4-CQA (Fig. 1a) compared to caffeoyl-isocitric acid (CIA) which eluted at Rt 9.6 min (Fig. 1d). Similarly, the same elution order was also consistent for *p*-coumaroyl-quinic acid (Fig. 1b) and feruloyl-quinic acid (Fig. 1c) with respect to their isocitric acid counterparts (Fig. 1e, f). Our results are consistent with the reported elution order observed elsewhere [25], where caffeoyl-quinic acids are seen to elute earlier than caffeoyl-isocitric acids on a C_18_ column. This suggests that in a reverse phase chromatographic space, the elution of IA conjugates is more retarded than the respective QA conjugates, an indication that IA derivatives are less polar than QA derivatives. This postulation can be explained by structural differences and stereochemistry of these compounds, resulting in differences in polarities. For instance, the QA possess more hydroxyl (–OH) groups (four in total), thus rendering it more polar relative to IA with only a single –OH group. Moreover, the IA has more C=O groups in close proximity which may led to the formation of intramolecular hydrogen bonds resulting in higher hydrophobicity. According to the experimentally determined LogP_o/w_ values shown elsewhere (http://www.chemspider.com), quinic acid is evidently more polar as it has a value of −2.01 whereas isocitric acid has a value of −1.47.

### Proposed fragmentation/structural re-arrangements of quinic- and isocitric acid

The results from MS analyses of hydroxycinnamoyl-quinic and hydroxycinnamoyl-isocitric acid show both QA and IA to be readily lost as product ions at *m/z* 191. However, the downstream MS fragmentation of these organic acids are different (Scheme 2). For instance, QA produces intense ions at *m/z* 191 [QA–H]^−^ and *m/z* 173, the latter resulting from the subsequent loss of water (−18 Da) [QA–H–H_2_O]^−^ (Scheme 2). Similarly, IA at *m/z* 191 also undergoes dehydration to give an ion at *m/z* 173 [IA–H–H_2_O]^−^. Consequently, the IA moiety undergoes further structural rearrangement when the ion at *m/z* 173 sequentially loses water (−18 Da) to give a unique ion at *m/z* 155 [IA–H–2H_2_O]^−^. The resulting product ion is further decarboxylated (−44 Da) to give another unique product ion at *m/z* 111 [IA–H–2H_2_O–CO_2_]^−^ (Scheme 2b). From the above it can be noted that the ions at *m/z* 155 and 111 characteristic for IA conjugates, which allows reliable distinction from QA derivatives.

## Conclusion

In conclusion, this work confirms the presence of hydroxycinnamoyl-isocitrates in *A. viridis* and hydroxycinnamoyl-quinates in *M. oleifera*, respectively. Although these compounds share similar MS molecular fingerprints, this work highlights the mass spectrometric fragmentation differences between the two related groups of compounds. Herein, the minor variations/differences with regard to the respective diagnostic peaks allow for the unambiguous annotation. As such, these findings illustrate the combinatorial and efficient ability of LC–MS to unequivocally distinguish between hydroxycinnamoyl-isocitrates and hydroxycinnamoyl-quinates. Furthermore, these findings are expected to provide a template reference for annotation of these compounds in other plants.

## Additional file

**Additional file 1. Figure S1.** Comparison of UPLC-SIM-MS chromatograms of selected HCA conjugates from surrogate standards of coffee (A and B) and pineapple extracts (C and D) and compared to *M. oleifera* and *A. viridis* extracts respectively. A Viva C_18_ analytical column (3.0 µm, 2.1 × 100 mm; Restek, USA) was eluted with a linear gradient at a constant flow rate of 400 µL/min of Methanol/Water mobile phase. The targeted ions were monitored using product ion scan MS/MS approach in ESI negative ionization mode at various collision energies (5–35 eV). A and B HCAs conjugated to quinic acid: (A) *p*-coumaroyl-quinic acid and (B) feruloyl-quinic acid. C and D HCAs conjugated to isocitric acid: (C) caffeoyl-isocitric acid and (D) *p*-coumaroyl-isocitric acid.
