# Supplementary material for: Highlighting mass spectrometric fragmentation differences and similarities between hydroxycinnamoyl-quinic acids and hydroxycinnamoyl-isocitric acids
Source: Chem Cent J. 2017 Apr 4;11:29. doi: 10.1186/s13065-017-0262-8 (PMC5380550; doi:10.1186/s13065-017-0262-8)
Supplement: Supplementary file 1 — Additional file 1. Figure S1. Comparison of UPLC-SIM-MS chromatograms of selected HCA conjugates from surrogate standards of coffee (A and B) and pineapple extracts (C and D) and compared to M. oleifera and A. viridis extracts respectively. A Viva C18 analytical column (3.0 µm, 2.1 × 100 mm; Restek, USA) was eluted with a linear gradient at a constant flow rate of 400 µL/min of Methanol/Water mobile phase. The targeted ions were monitored using product ion scan MS/MS approach in ESI negative ionization mode at various collision energies (5–35 eV). A and B HCAs conjugated to quinic acid: (A) p-coumaroyl-quinic acid and (B) feruloyl-quinic acid. C and D HCAs conjugated to isocitric acid: (C) caffeoyl-isocitric acid and (D) p-coumaroyl-isocitric acid. [file 13065_2017_262_MOESM1_ESM.docx]

Supplementary Data 1


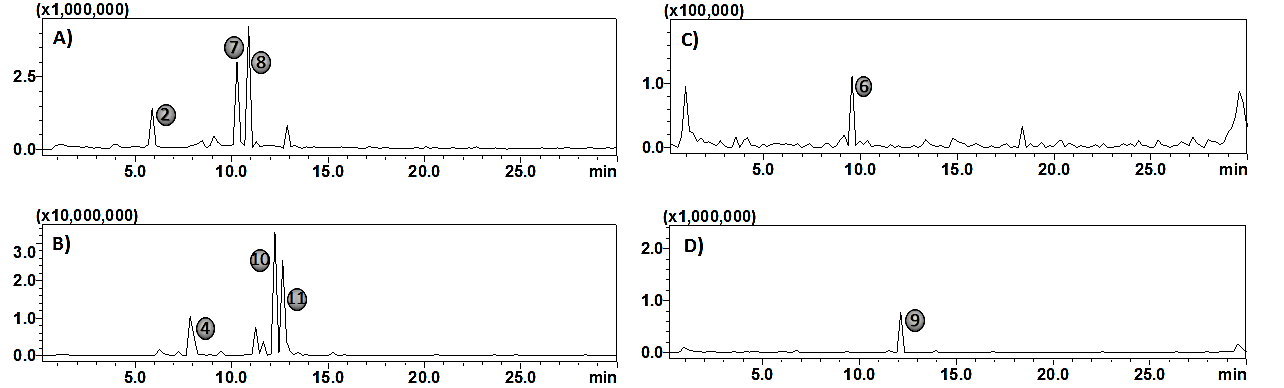


Figure S1: Comparison of UPLC-SIM-MS chromatograms of selected HCA conjugates from surrogate standards of coffee (**A** and **B**) and pineapple extracts (**C** and **D**) and compared to *M. oleifera* and *A. viridis* extracts respectively. A Viva C_18_ analytical column (3.0 µm, 2.1 × 100 mm; Restek, USA) was eluted with a linear gradient at a constant flow rate of 400 µL/min of Methanol/Water mobile phase. The targeted ions were monitored using product ion scan MS/MS approach in ESI negative ionization mode at various collision energies (5 - 35 eV). **A** and **B** HCAs conjugated to quinic acid: **(A)** *p*-coumaroyl-quinic acid and **(B)** feruloyl-quinic acid. **C** and **D** HCAs conjugated to isocitric acid: **(C)** caffeoyl-isocitric acid and **(D)** *p-*coumaroyl-isocitric acid.
